# Aberrant generation of dentate gyrus granule cells is associated with epileptic susceptibility in p53 conditional knockout mice

**DOI:** 10.3389/fnins.2024.1418973

**Published:** 2024-08-14

**Authors:** Nuria Ruiz-Reig, Georges Chehade, Xavier Yerna, Irene Durá, Philippe Gailly, Fadel Tissir

**Affiliations:** ^1^Laboratory of Developmental Neurobiology, Institute of Neuroscience, Université catholique de Louvain, Brussels, Belgium; ^2^Laboratory of Cell Physiology, Institute of Neuroscience, Université catholique de Louvain, Brussels, Belgium; ^3^College of Health and Life Sciences, Hamad Bin Khalifa University, Doha, Qatar

**Keywords:** Trp53, adult neurogenesis, epileptic seizures, hippocampus, subgranular zone

## Abstract

Neuronal apoptosis is a mechanism used to clear the cells of oxidative stress or DNA damage and refine the final number of neurons for a functional neuronal circuit. The tumor suppressor protein p53 is a key regulator of the cell cycle and serves as a checkpoint for eliminating neurons with high DNA damage, hyperproliferative signals or cellular stress. During development, p53 is largely expressed in progenitor cells. In the adult brain, p53 expression is restricted to the neurogenic niches where it regulates cell proliferation and self-renewal. To investigate the functional consequences of p53 deletion in the cortex and hippocampus, we generated a conditional mutant mouse (p53-cKO) in which p53 is deleted from pallial progenitors and their derivatives. Surprisingly, we did not find any significant change in the number of neurons in the mutant cortex or CA region of the hippocampus compared with control mice. However, p53-cKO mice exhibit more proliferative cells in the subgranular zone of the dentate gyrus and more granule cells in the granular cell layer. Glutamatergic synapses in the CA3 region are more numerous in p53-cKO mice compared with control littermates, which correlates with overexcitability and higher epileptic susceptibility in the mutant mice.

## 1 Introduction

The final number of neurons in the mature brain is crucial for the proper functioning of the neuronal circuitry. In physiological conditions, the nervous system overproduces neuronal cells and subsequently eliminates neurons that are not functionally integrated in the neural networks (Yamaguchi and Miura, 2015). There are two waves of cell death in the mouse brain. The first occurs during embryonic development whereas the second takes place during the first two postnatal weeks (Wong and Marin, 2019). Intrinsic apoptosis, one of the mechanisms of programmed cell death in the central nervous system, involves activation of proapoptotic proteins such as Bax and Bak (Wei et al., 2001). Ninety percent of mice with genetic deficiency in Bax and Bak (*Bax^−/−^; Bak^−/−^* double mutant mice) die perinatally. The mutant mice that survive to adulthood exhibit a larger brain and higher number of neurons (Lindsten et al., 2000). In the cortex, programmed cell death starts from E10.5 and affects neuronal progenitors and early neurons during development. In the second wave at the early postnatal stage, pyramidal neurons and GABAergic interneurons undergo cell death to adjust their final number. Other cell populations such as Cajal–Retzius cells, subplate neurons and early born oligodendrocytes are completely eliminated (Wong and Marin, 2019).

During embryonic stages, p53 regulates neural tube closure and the production of neural cells (Armstrong et al., 1995). p53 regulates the cell cycle and acts as a checkpoint in the elimination of cells with high oxidative stress or DNA damage (Jacobs et al., 2006). From E10.5 to E16.5, p53 is expressed in neural progenitors in the ventricular zone (VZ) (Schmid et al., 1991) where it is believed to promote neurogenesis at the expense of gliogenesis (Armesilla-Diaz et al., 2009; Forsberg et al., 2013; Liu et al., 2013). By interacting with Bax and Bak proteins and activating the apoptotic program, the tumor suppressor protein p53 may control cell survival (Chipuk et al., 2004; Leu et al., 2004). However, the role of p53 in programmed cell death is debated (Miller et al., 2000). p53 is also required for NGF-mediated neurite extension via Kif2a activation, a microtubule depolymerizing protein implicated in neuronal morphology (Di Giovanni et al., 2006; Tedeschi et al., 2009; Sun et al., 2010; Ruiz-Reig et al., 2022, 2024).

At postnatal stages, p53 is expressed in the neurogenic niches and neuroblasts migrating through the rostral migratory stream (van Lookeren Campagne and Gill, 1998; Meletis et al., 2006). In the adult subventricular zone (SVZ), *p53^−/−^* mice have a higher number of proliferative cells associated with an elevated proliferation and self-renewal of type B (transient amplifying precursors) and type A (neuroblasts) cells (Gil-Perotin et al., 2006; Meletis et al., 2006). That is translated into more newborn neurons in the olfactory bulb (OB) in *p53^−/−^* mice (Gil-Perotin et al., 2011). Adult neurogenesis also occurs in the hippocampus of mice, where progenitors in the subgranular zone (SGZ) generate newborn granule cells that integrate into the dentate gyrus. Changes in hippocampal adult neurogenesis have been associated with epilepsy in mice (Jung et al., 2004, 2006; Cho et al., 2015; Hosford et al., 2017). Interestingly, the role of p53 in adult neurogenesis, and its relationship with epileptic susceptibility has not been investigated. In this study, we specifically ablated p53 from pallial progenitors and their derivatives in the dorsal telencephalon and studied the functional consequences on cortical and hippocampal development and function. Conditional p53 mutant mice (p53-cKO) do not exhibit any glaring phenotype in the neocortex, suggesting that p53 is dispensable for cortical development. However, p53-cKO mice have altered hippocampal neurogenesis and are more vulnerable to epileptic seizures. Consistent with the role of p53 in proliferation in the adult SVZ, we found an increase in subgranular Sox2+ progenitors in the adult hippocampus of p53-cKO mice, which was associated with a rise in the number of granule cells in the dentate gyrus and density of synapses in the CA3 region. Electrophysiological recording in CA3 of the mutant hippocampus revealed an overexcitability after the blocking of GABA_A_ receptors with picrotoxin, suggesting a higher inhibitory state in the mutant hippocampus. Overall, this study revealed a link between p53, adult neurogenesis in the hippocampus, and susceptibility to epileptic seizures in mice.

## 2 Materials and methods

### 2.1 Mice

All animal procedures were carried out following European guidelines (2010/63/UE) and approved by the animal ethics committee of the Université Catholique de Louvain under agreement 2019/UCL/MD/006. The following mouse lines were used: *Emx1-Cre* (Gorski et al., 2002), and *Trp53^F/F^* (Marino et al., 2000). To produce *Emx1-Cre*; *Trp53^F/F^* mice (p53-cKO), we crossed *Emx1-Cre; Trp53^F/F^* females with *Trp53^F/F^* males. *Trp53^F/F^* littermates (without Cre) were considered as control mice. All experiments were carried out on both males and females without any distinction of gender.

### 2.2 PTZ-induced epileptic seizures

To evaluate epileptic susceptibility in mice, experiments were performed as described in Shimada and Yamagata (2018). PTZ solution (Sigma P6500, 10 mg/mL in sterile 0.9% NaCl) was prepared freshly on the day of use. 8-weeks-old mice were placed in an observation cage for a 3-min habituation period and then injected with an intraperitoneal single dose of PTZ (dose 35 mg/Kg). Following injection, mice behavior was monitored for 30 min and classified according to the following scoring: 0: normal behavior, no abnormality; 1: immobilization, lying on belly; 2: head nodding, facial, forelimb, or hindlimb myoclonus; 3: continuous whole-body myoclonus, myoclonic jerks, tail held up stiffly; 4: rearing, tonic seizure, falling down on its side; 5: tonic–clonic seizure, falling down on its back, wild rushing and jumping; 6: death (Shimada and Yamagata, 2018). Mice were injected every other day three times. All the injections were performed in the same room and in the morning, before noon.

### 2.3 Immunofluorescence and *in situ* hybridization

Adult mice were intracardially perfused with PBS and 4% paraformaldehyde (PFA) in 0.1 M phosphate buffer (PB), pH 7.4, and with PFA 4%, in 0.1 PBS pH 7.4 or PFA 4%, picric acid 15% in 0.1 M PB for synaptic staining (Vglut1, PSD95, Vgat and GABAAα2). Brains were harvested and post-fixed in the same fixative for 2 h at room temperature (RT) for immunohistochemistry and overnight (ON) at 4°C for *in situ* hybridization (ISH). Postnatal brains were washed in PBS, embedded in 4% agarose, and sectioned with a Leica VT1000S vibratome (40 μm). Immunohistochemical staining was performed as previously described (Ruiz-Reig et al., 2017). We used the following primary antibodies: Rabbit anti-Foxp2 (Abcam ab16046, 1:500); Rabbit anti-Cux1 (Proteintech 11,733-1-AP, 1:250); Rat anti-Ctip2 (AbCam ab18465, 1:250); Rabbit anti-Calbindin D-28 K (Swant CB38, 1:2000); Rabbit anti-Sox2 (Millipore ab5603, 1:200); Mouse anti-Ki-67 (BD Pharmingen 556,003, 1:50); Goat anti-Prox1 (R&D AF2727, 1:100); Guinea pig anti-Vglut1 (Millipore AB5905, 1:1000); Mouse anti-PSD95 (Thermofisher MA1-045, 1:250); Mouse anti-Vgat (Synaptic Systems 131,011, 1:1,000); Rabbit anti-GABAAα2 (Synaptic Systems 224,103, 1:500). Different AlexaFluor-conjugated secondary antibodies (Invitrogen, 1:800) were used. After immunohistochemistry, the sections were incubated with DAPI (Sigma D9564 100 μM) for 5 min and mounted with Mowiol. ISH as described in Ruiz-Reig et al. (2018). Brain vibratome sections were hybridized with a biotinylated Gad67 riboprobe from the Gad67 plasmid (van den Berghe et al., 2013). Sections were dehydrated in ethanol, incubated twice in toluene for 10 min, and mounted with Neo-Mount® medium (Merck 109,016). To evaluate cell death, we used TUNEL staining (*In situ* Cell Death Detection Kit, TMR red; Roche) on vibratome sections. As positive control, we used sections pretreated with DNAseI (1,000 U/mL) for 10 min at room temperature.

### 2.4 Brain slice preparation

Brain slice preparation was performed as previously described (Yerna et al., 2020). Briefly, animals were sacrificed by cervical dislocation and their brains were quickly removed and placed in ice-cold artificial cerebrospinal fluid (ACSF) composed of (mM): 126 NaCl, 3 KCl, 2.4 CaCl2, 1.3 MgCl2, 1.24 NaH2PO4, 26 NaHCO3, and 10 glucose (bubbled with 95%O2-5% CO2%). Cerebellum and frontal cortex were removed. Brains were mounted onto a LeicaVT1200 vibratome, and horizontal sections of a thickness of 350 mm were cut in ice-cold ACSF to obtain ventral hippocampus. Slices recovered in oxygenated ACSF at 32°C for at least 1 h before use.

### 2.5 Field potential recordings

**Figure 1:**
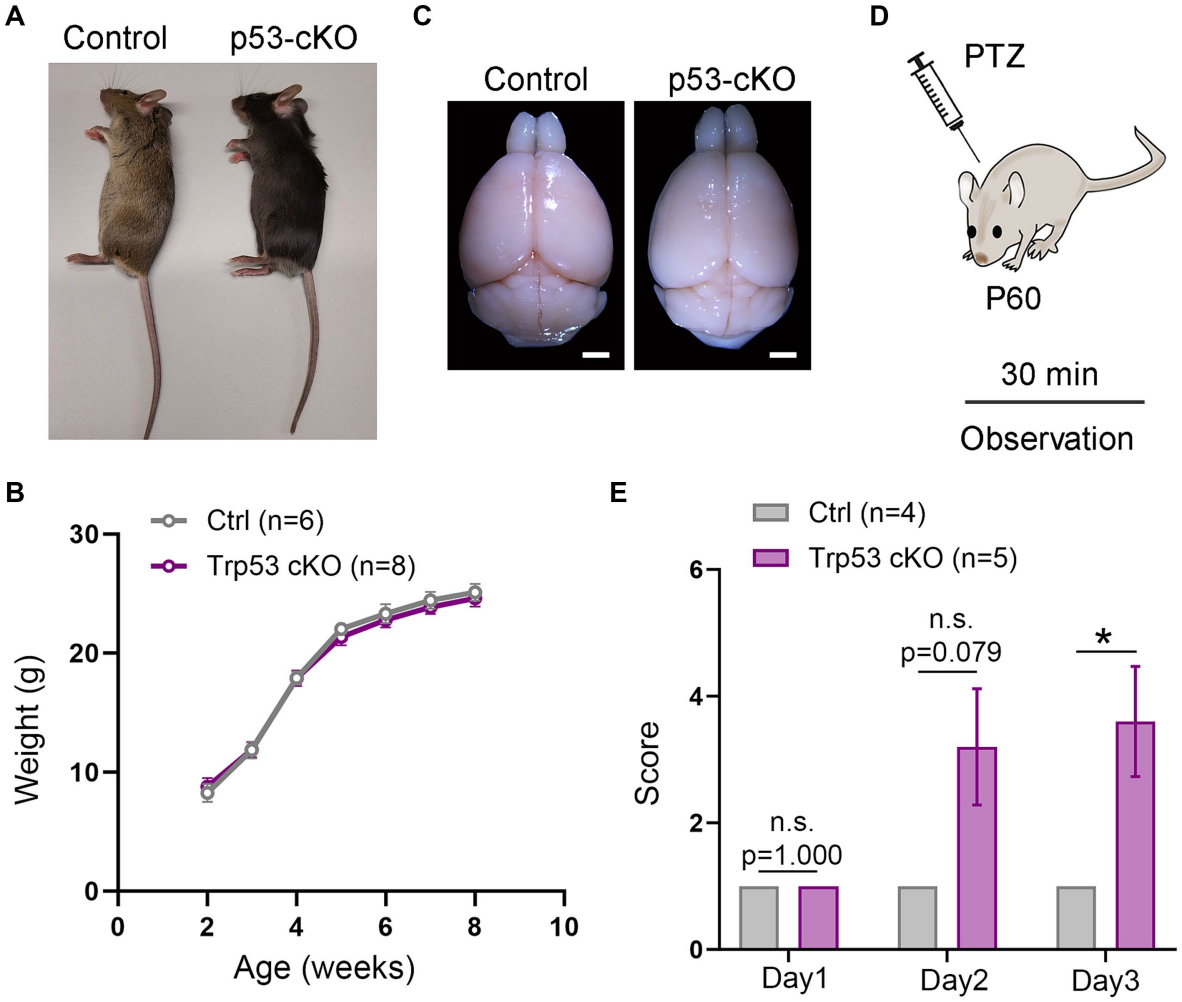
Epileptic susceptibility in p53-cKO mice. **(A,B)** No differences were observed in terms of size **(A)** and weight **(B)** between control and p53-cKO mice up to 2 months. **(C)** Mutant brains are macroscopically similar to control brains. **(D)** In order to analyze epileptic susceptibility, 2-month-old mice (P60) were injected with Pentylenetetrazol (PTZ), and their behavior was observed for 30 min. **(E)** Increased epileptic susceptibility was observed from the second day of injection (Ctrl: 1 *n* = 4; p53-cKO: 3.2 ± 0.9 *n* = 5) and significantly different in the third day of injection (Ctrl: 1 *n* = 4; p53-cKO: 3.6 ± 0.87 *n* = 5; *p* = 0.034). Values were obtained by unpaired Student’s *t*-test; *p < 0.05, **p < 0.01, and ***p < 0.001. Scale bar **(A)** 1.7 mm.

Field Potential Recordings were performed as previously described (Lepannetier et al., 2018). Briefly, mouse brain slices were transferred to the recording chamber and continuously perfused with oxygenated ACSF (2 mL/min) at 30°C. Excitatory postsynaptic potentials (EPSP) and population spikes (PS) were evoked through a bipolar stimulating electrode which was placed in the hilus region close to the dentate granule cell layer to stimulate mossy fibre axons (Figure 1A). Responses were recorded by the AxoClamp 2B (Axon Instruments, United States) amplifier through a glass electrode which was back-filled with 2 M NaCl and placed in the CA3 region (stratum lucidum or stratum pyramidale for EPSP and PS recording respectively). Stimuli consisted of 100 μs pulses of constant currents. Responses were digitized by Digidata 1322A (Axon Instruments, United States) and recorded to a computer using WinLTP software (Anderson and Collingridge, 2007).

### 2.6 Data analysis and image processing

Images were captured with a digital camera coupled with an inverted Zeiss Axio Observer microscope or in a Laser Scanning confocal microscope (Olympus Fluoview FV1000). Figures were prepared using Adobe Photoshop and Adobe Illustrator CC 2019, and 2D mosaic reconstructions were produced when needed using the Photomerge tool of Photoshop software package. Cell counting was conducted manually using Fiji software (ImageJ). For Sox2 and Ki-67 positive cells in the dentate gyrus, the images were taken with a confocal microscope with a 20X objective and 10 μm z (1 μm step size). Image stack were reconstructed and analyzed with Fiji software to calculate Sox2 and Ki-67 positive cells in the subgranular zone of the dentate gyrus. For Vglut1+/PSD95+ and GABAAα2+/Vgat+ clusters in the hippocampus, confocal images were obtained with a 63x objective and 3x digital zoom. Image stacks (0.5 μm step size) were reconstructed and analyzed with Fiji software to calculate Vglut1+/PSD95+ and GABAAα2+/Vgat+ cluster density in the CA3 region of the hippocampus. Counting was performed in a 625 μm2 area and quantification was normalized with the control samples. All the cell quantifications were performed in a minimum of three different sections per animal and represented as the mean value per animal. Quantification of data and graphs were constructed using Prism 9 (GraphPad, San Diego, CA, United States) software. A minimum of 3 animals per genotype was used for all analyses and quantifications. The exact sample size is specified in the result section or figure legends. Error bars represent the standard error of the mean (SEM). We performed a Shapiro-Wilks test to evaluate the distribution of the data. We then used a two-tailed Student’s t-test when the data followed a normal distribution and Mann–Whitney test when it did not (n.s. not significant, *p < 0.05, **p < 0.01, ***p < 0.001). Observations without quantification have been validated and successfully reproduced in a minimum of three different animals.

## 3 Results

### 3.1 Conditional deletion of p53 in the pallium of mice triggers epileptic seizures

To study the functional consequences of p53 deletion in cortical development and function, we designed a conditional KO mouse line in which p53 is deleted only from pallial progenitors and their derivatives from E9.5 (*Emx1^Cre^ Trp53^F/F^* called hereafter p53-cKO) and we compared it with control littermates (*Trp53^F/F^*). Mutant mice were similar in size and weight to control littermates (Figures 1A,B) and their brains were macroscopically undistinguishable (Figure 1C). However, we noted that p53-cKO mice develop spontaneous seizures starting from 1 month (Supplementary Video S1). To test whether mutant mice are more susceptible to developing epileptic seizures under pharmacological induction, we injected Pentylenetetrazol (PTZ), a GABA_A_ receptor antagonist, during three alternate days and scrutinized mice behavior for 30 min (Figure 1D). Control mice exhibit immobilization (score 1) but did not display seizure on any analyzed day (Figure 1E; Supplementary Video S2). In contrast, 4 out 5 p53-cKO mice showed signs of epilepsy seizures already starting from the second injection (score between 2 and 5, Figure 1E). This data indicates that the conditional deletion of p53 in pallial progenitors and glutamatergic neurons of the cortex and hippocampus triggers epileptic seizures in mice.

### 3.2 No cortical development abnormalities in p53 conditional mutant mice

**Figure 2:**
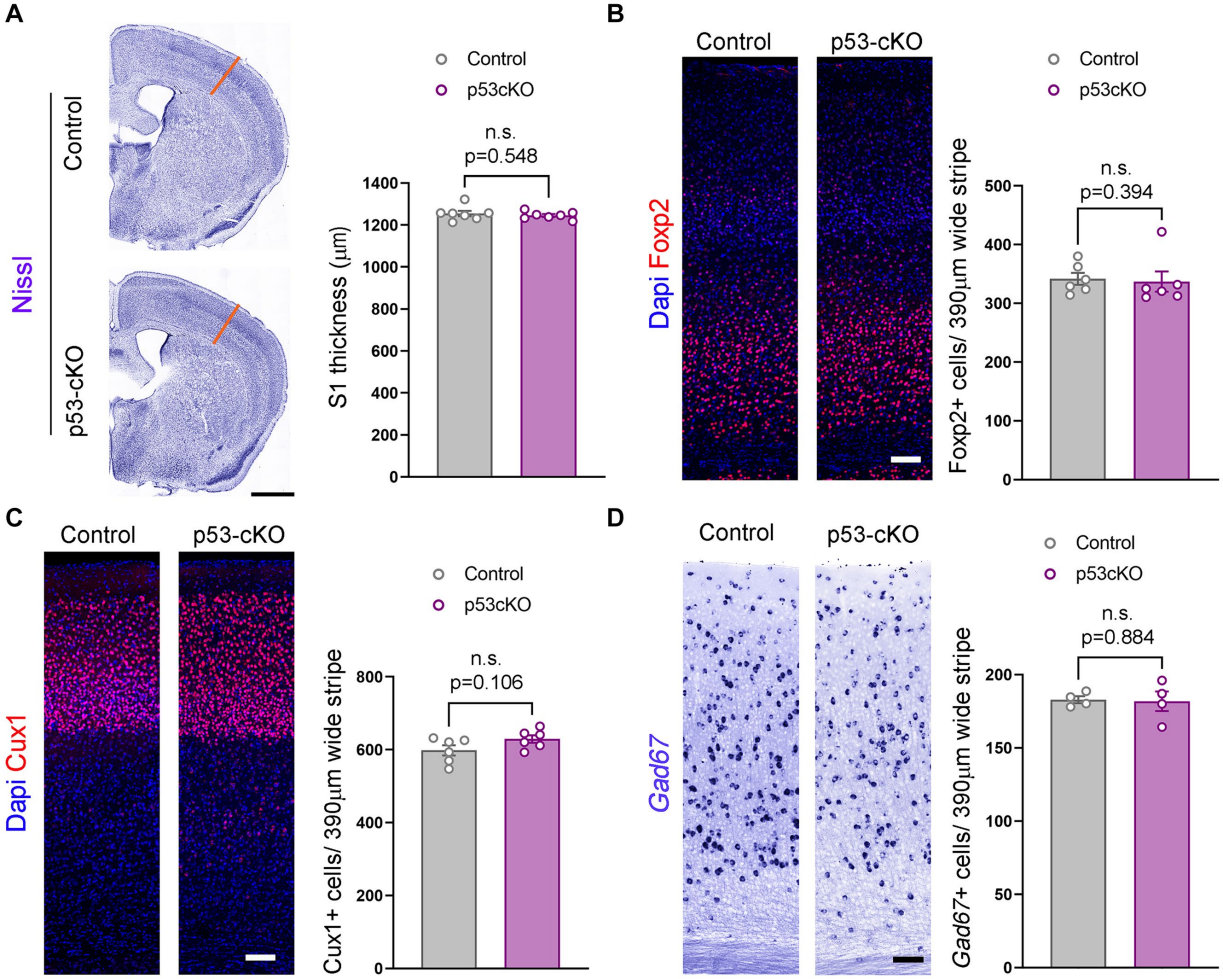
Similar cortical cytoarchitecture in p53-cKO mutant mice. **(A)** Coronal sections labeled with Nissl staining (left) and quantification of the thickness of the somatosensory area (right) (S1, orange lines). **(B)** Immunofluorescence for Foxp2 (left) and mean density of Foxp2+ cells in the S1 area (right) of the indicated genotypes at P60. **(C)** Immunofluorescence for Cux1 (left) and mean density of Cux1+ cells in the S1 area (right) of the indicated genotypes at P60 **(D)** Coronal sections at the level of the S1 area hybridized with *Gad67* riboprobe in control and p53-cKO mice (left) and mean density of *Gad67*+ cells in the S1 area (right) of the indicated genotypes at P60. Values were obtained by unpaired Student’s *t*-test for **(C)** and **(D)** and Mann -Whitney test for Foxp2 quantification **(B)**; *p < 0.05, **p < 0.01, and ***p < 0.001. Scale bar **(A)** 1 mm, **(B–D)** 100 μm.

To explore whether p53 contributes to programmed cell death in the cortex, we analyzed the thickness of the somatosensory cortex (S1) and did not detect any noticeable difference (Ctrl = 1,255 ± 12.8 μm *n* = 7; p53-cKO = 1,246 ± 7 μm *n* = 7; *p =* 0.548, Figure 2A). To analyze the potential implication of p53 deletion in cortical layering, we used Foxp2 (a marker of deep cortical neurons) and Cux1 (upper cortical neurons) immunofluorescence and did find any significant difference between the two genotypes (number of Foxp2-positive neurons: Ctrl = 341.7 ± 10.18 cells/390 μm wide stripe *n* = 6, p53-cKO = 337 ± 17.24 cells/390 μm wide stripe *n* = 6, *p =* 0.394, Figure 2B; number of Cux1-positive neurons; Ctrl = 598.4 ± 13.97 cells/390 μm wide stripe *n* = 6, p53-cKO = 629.4 ± 10.48/390 μm wide stripe *n* = 6, *p =* 0.106, Figure 2C). Although we deleted p53 only in glutamatergic progenitors and neurons, we analyzed the number of cortical interneurons in the cortex, since the final number of GABAergic neurons depends on neuronal activity. Here again, we did not detect any change in the density of GAD67+ positive neurons in the S1 cortical region (Ctrl = 182.9 ± 2.36 cells/390 μm wide stripe *n* = 4; p53-cKO = 181.8 ± 6.7/390 μm wide stripe *n* = 4, *p =* 0.884, Figure 2D). These results indicate that p53 deletion in the pallium does not alter the cytoarchitecture of the neocortex.

### 3.3 p53 Deletion increases granule cell generation in adult mice and its distribution in the dentate gyrus

**Figure 3:**
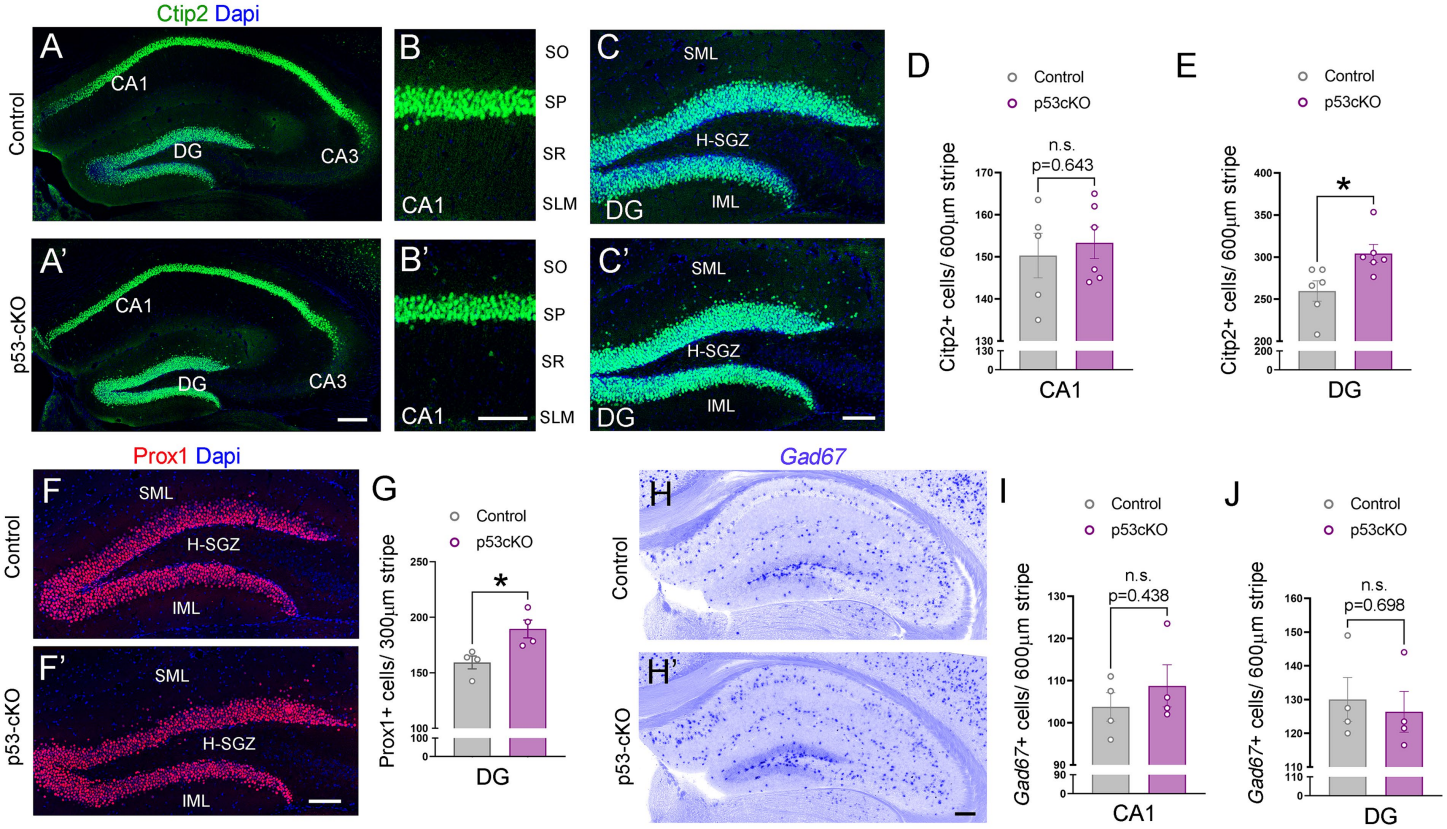
Increased granule cell density in p53-cKO hippocampus. **(A)** Immunofluorescence for Ctip2 in coronal sections at the level of the hippocampus of control and p53-cKO mice at P60. **(B,B′)** Magnification of the CA1 region depicting Citp2-positive cells in control **(B)** and p53-cKO mice **(B′)**. **(C,C′)** Magnification of the DG depicting Citp2-positive cells in control **(C)** and p53-cKO mice **(C′)**. **(D,E)** Quantification of Ctip2 density in the CA1 region **(D)** and DG **(E)**. (**F,F′**) Immunofluorescence for Prox1 on coronal sections at the level of the DG of control and p53-cKO mice at P60. (**G**) Quantification of Prox1 positive cells density in the DG **(H,H′)** Hippocampal coronal sections hybridized with *Gad67* riboprobe in control **(F)** and p53-cKO mice **(F′)**. **(I,J)** The mean density of *Gad67*-positive neurons in the CA1 region **(G)** and DG **(H)**. DG, dentate gyrus; SO, stratum oriens; SP, stratum pyramidale; SR, stratum radiatum; SLM, stratum lacunosum-moleculare; SML, superior molecular layer; IML, inferior molecular layer; H-SGZ, Hilus-subgranular zone. Values were obtained by unpaired Student’s *t*-test; *p < 0.05, **p < 0.01, and ***p < 0.001. Scale bar **(A’,H′)** 200 μm **(B′,C′,F′)** 100 μm.

**Figure 4:**
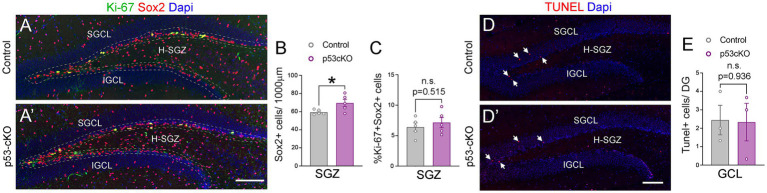
The lack of p53 increases the number of Sox2+ progenitors in the hippocampus. **(A,A’)** Coronal section immunolabeled with Sox2 and Ki-67 antibodies at the level of the dentate gyrus of control **(A)** and p53-cKO mice **(A’)** at P60. The dashed line delineated the SGZ of the hippocampus. **(B)** Quantification of the density of Sox2 positive cells in the SGZ of the hippocampus. **(C)** Quantification of Sox2 and Ki-67 positive cells among the Sox2 positive population in the SGZ. **(D,D′)** Coronal sections at the level of the DG stained with TUNEL in control **(D)** and mutant mice **(D′)**. **(E)** Quantification of TUNEL positive cells in the GCL per dentate gyrus. GCL, granula cell layer; SGCL, superior granular cell layer; IGCL, inferior granular cell layer; H-SGZ, Hilus-subgranular zone. Values were obtained by unpaired Student’s *t*-test; *p < 0.05, **p < 0.01, and ***p < 0.001. Scale bar **(A’,D′)** 100 μm.

To investigate possible anatomical abnormalities in the hippocampus due to p53 deletion, we scrutinized the cellular composition of hippocampal formation. We labeled pyramidal neurons of the CA1 and granular neurons of the dentate gyrus (DG) with Ctip2 and Prox1 antibodies (Figures 3A,A’). While the number of Ctip2 positive neurons in the CA1 region was unaffected by the loss of p53 (Ctrl = 150 ± 5.3 cells/600 μm wide stripe *n* = 5, p53-cKO = 153.3 ± 3.7/600 μm wide stripe *n* = 6; *p =* 0.643, Figures 3B,B’,D), the number of granule cells labeled with Ctip2 and Prox1 was significantly greater in p53-cKO mice compared with control littermates (Ctip2: Ctrl = 259.7 ± 12 cells/600 μm wide stripe *n* = 6, p53-cKO = 304.3 ± 10.6/600 μm wide stripe *n* = 6; *p =* 0.019, Prox1: Ctrl = 159.4 ± 5.8 cells/300 μm wide stripe *n* = 4, p53-cKO = 189.5 ± 8/300 μm wide stripe *n* = 4; *p =* 0.023, Figures 3C,C’,E, F,F’,G). Importantly, some granule cells were scattered in the superior molecular layer (SML) of the mutant dentate gyrus (Figures 3C,C’,F,F’) but we did not find Ctip2 or Prox1 positive cells in the hilus of the mutant hippocampus. We did not observed any difference in the number of *Gad67*-positive cells in the CA1 or DG of the hippocampus between control and mutant mice (CA1: Ctrl = 103.8 ± 3.4 cells/600 μm wide stripe *n* = 4, p53-cKO: 108.8 ± 5/600 μm wide stripe *n* = 4, *p =* 0.438; DG: Ctrl:=130 ± 6.5 cells/600 μm wide stripe *n* = 4, p53-cKO = 126.4 ± 6.4/600 μm wide stripe *n* = 4; *p =* 0.698, Figures 3H–J). To assess the role of p53 in the proliferation and self-renewal of progenitors in the SVZ of adult mice, we analyzed the number of progenitors Sox2-positive in the subgranular zone (SGZ) (Figures 4A,A’). We observed a significantly higher density of these progenitors in the mutant DG compared with control littermates (Ctrl = 59.35 ± 1.2 cells/1000 μm *n* = 5; p53-cKO = 69.49 ± 4/1000 μm *n* = 5, *p =* 0.043, Figure 4B). Some of the Prox1cells in the SVZ were positive for Ki-67 (Figures 4A,A’). The percentage of cells positive for Ki-67 and Sox2 among the Sox2 population in the SGZ was not change between Ctrl and mutant mice (Ctrl = 6.4 ± 0.69 *n* = 5; p53-cKO = 7.1 ± 0.84 *n* = 5; *p =* 0.515; Figures 4A,A’,C). We did not find a decrease in the number of TUNEL-positive cells in the mutant hippocampus indicating no change in cell death (Ctrl = 2.44 ± 0.8 *n* = 3; p53-cKO = 2.33 ± 1 *n* = 3; *p = 0.936* Figures 4D,D’,E). These results suggest that p53 negatively regulates adult neurogenesis in the hippocampus and p53 deletion increases the number of adult progenitors and granule cells in the dentate gyrus.

### 3.4 p53 Mutant mice have increased glutamatergic synapses in the hippocampus

**Figure 5:**
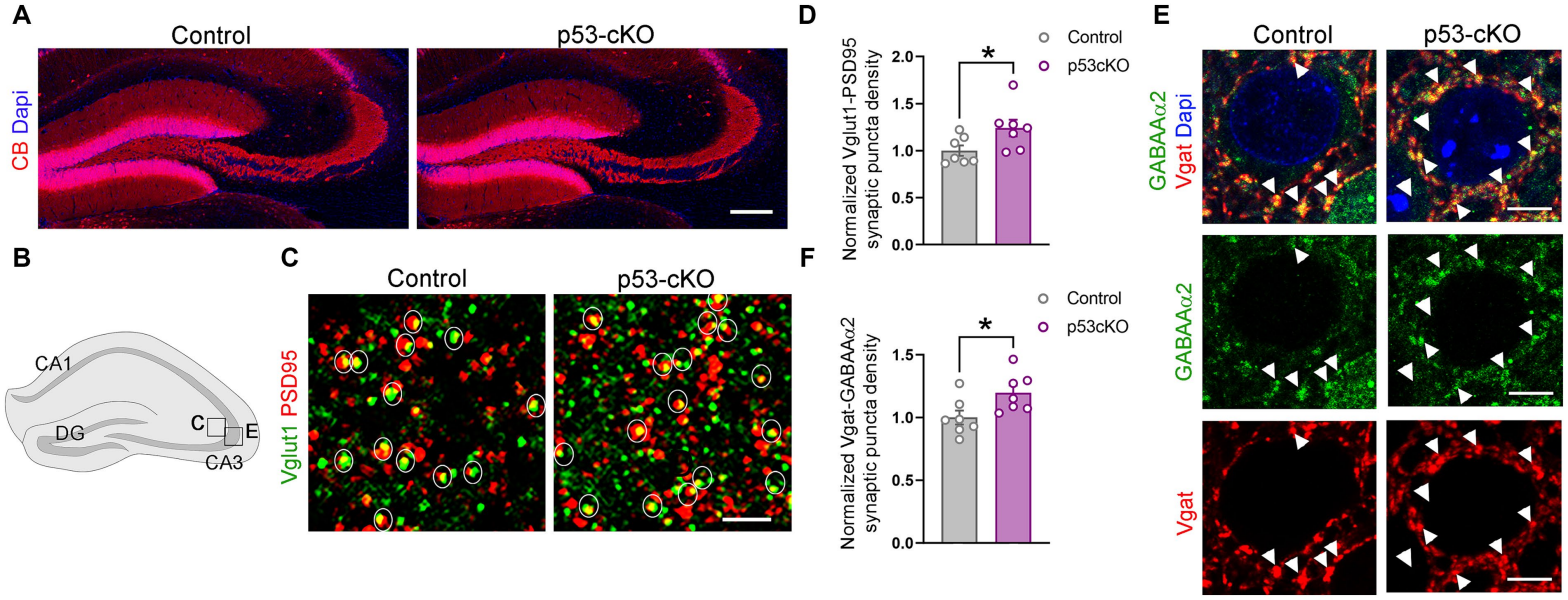
p53-cKO mice present an increase in glutamatergic and GABAergic synapses in the CA3 region. **(A)** Immunofluorescence for Calbindin (CB) in coronal sections at the level of the hippocampus of control and p53-cKO mice at P60 labeling the granule cells of the dentate gyrus. **(B)** Schematic illustration of a hippocampus showing the regions where the pictures were taken. **(C)** Immunofluorescence for Vglut1 and PSD95 in the stratum lucidum of the CA3 region. Circles delineated the synaptic puncta (colocalization between Vglut1 and PSD95). **(D)** Quantification of the density of glutamatergic synaptic puncta in the CA3 region of control and p53-cKO mice (Ctrl: 1 ± 0.06, *n* = 7; p53-cKO: 1.24 ± 0.09, *n* = 7). **(E)** Immunofluorescence for GABAAα2 and Vgat in the stratum pyramidale of the CA3 region. Arrowheads indicate the GABAergic synaptic puncta (colocalization between GABAAα2 and Vgat). **(F)** Quantification of the density of GABAergic synaptic puncta in the CA3 region of control and p53-cKO mice (Ctrl: 1 ± 0.06, *n* = 7; p53-cKO: 1.2 ± 0.06, *n* = 7). DG, dentate gyrus. Values were obtained by unpaired Student’s *t*-test; *p < 0.05, **p < 0.01, and ***p < 0.001. Scale bar **(A)** 200 μm, **(C)** 2 μm, **(E)** 5 μm.

Mossy fiber sprouting is one of the reasons for the epileptic brain’s increased excitability. Calbindin staining did not detect any aberrant growth of the dentate gyrus granule cell axons (Figure 5A). However, analysis of the density of glutamatergic synapses (Vglut1 + Psd95+ puncta) in the stratum lacosum of the CA3 region (Figures 5B,C), revealed more excitatory puncta in p53 cKO compared with control mice (+24% ± 0.1; *p =* 0.042, Figures 5C,D). Interestingly, although we did not find changes in the number of GABAergic interneurons in the mutant hippocampus, we observed an increase in the number of inhibitory synapses (Vgat+GABAAα2+) around the soma of CA3 pyramidal neurons in the stratum pyramidale (+19.7% ± 0.08; *p =* 0.031, Figures 5B,E,F). These results indicate that p53-cKO mice present an increase in excitatory synapses with a concomitant increase in inhibitory synapses around the soma of pyramidal neurons in the CA3 region of the hippocampus.

### 3.5 Overexcitability in p53 mutant hippocampus after picrotoxin perfusion

**Figure 6:**
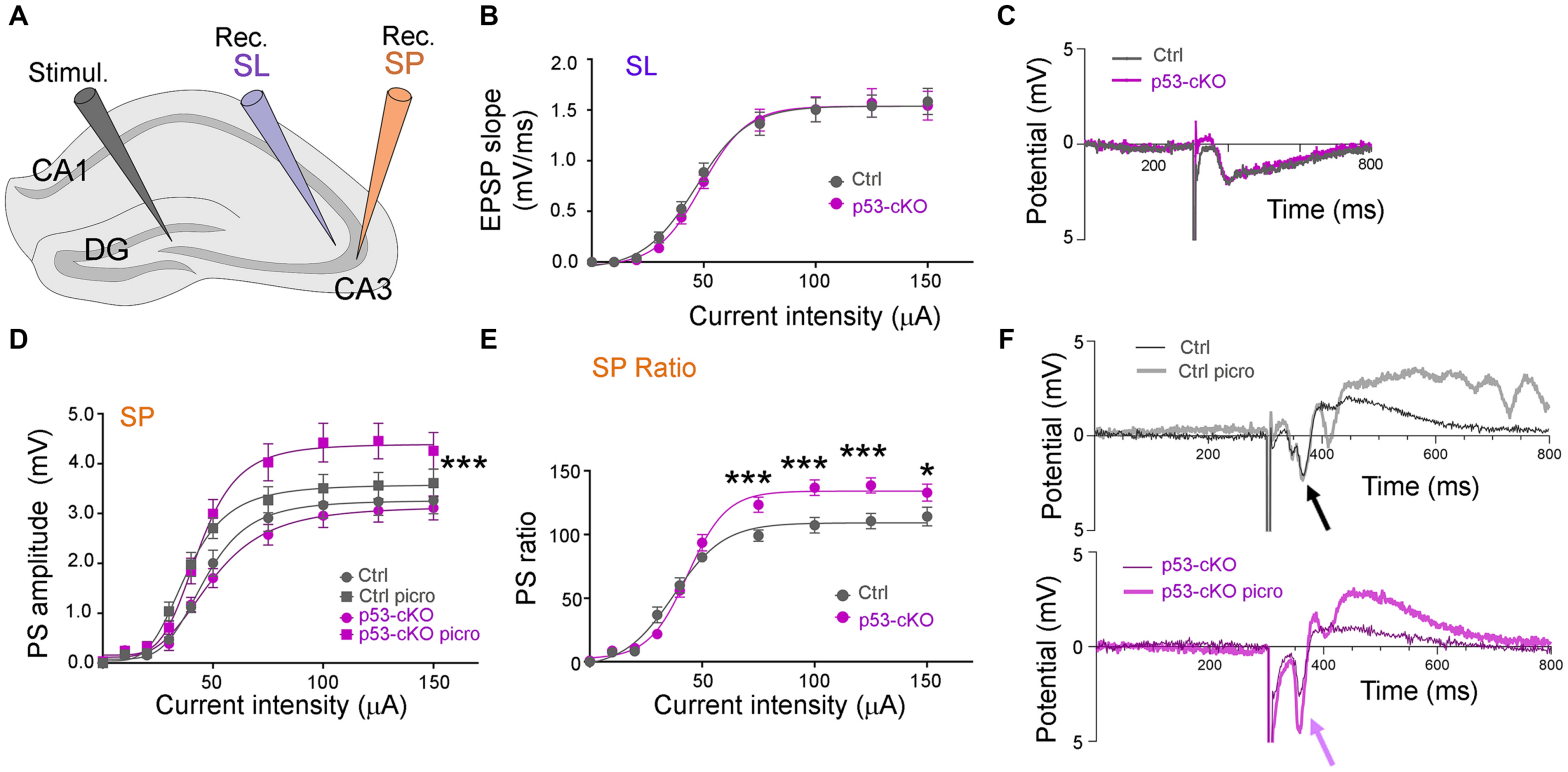
Basal DG-CA3 synaptic transmission in p53-cKO compared to control mice. **(A)** Disposition of the stimulation (Stimul.) and the recording (Rec.) electrodes in brain hippocampal slices. **(B)** The I/O relationship between the intensity of DG stimulation and the fEPSP slope was measured in CA3 stratum lucidum (SL). **(C)** Examples of fEPSP recording traces. **(D)** The I/O relationship between the intensity of DG stimulation and the PS amplitude measured in CA3 stratum pyramidale (SP), before and after 10 min stimulation with 100 μM picrotoxin (Three-way ANOVA with Tukey’s multiple comparison test, *** *p* < 0.001; *n* = 21 for Ctrl and 20 for p53-cKO). **(E)** Ratios of the responses before and after picrotoxin stimulation, in Ctl and cKO brain slices (Mann–Whitney test *p < 0.05; ***p < 0.001; *n* = 21 for Ctrl and 20 for p53 cKO). **(F)** Examples of PS recording traces. Note the effect of picrotoxin in p53-cKO slices (purple arrow) and the absence of effect in Ctrl (black arrow).

To test whether p53 deletion affects basic synaptic transmission, we measured the input/output (I/O) functions in the hippocampus of control and p53-cKO mice (Figure 6A). The I/O curve of Exctitatory PostSynaptic Potential (EPSP) slopes was first measured in the stratum lucidum and was similar in both genotypes (Figures 6B,C). We then recorded in the stratum pyramidale and measured postsynaptic (PS) amplitude (Figure 6D). Again, no statistically significant difference was observed between control and p53 cKO mice. Thereafter, without moving the recording electrode, we perfused the slice for 10 min with 100 μM picrotoxin, an inhibitor of GABAAR. Picrotoxin induced a slight increase of the PS responses and slightly left-shifted the response curves, the I50 (the intensity giving 50% of the maximal response, measured by nonlinear regression curve fitting with variable slope) passing from 46.2 μA to 38.2 μA in Ctrl (*p =* 0.007, *n* = 21) and from 50.8 μA to 43.5 μA in p53 cKO (*p =* 0.059, *n* = 20; Figure 6D). The PS responses were significantly more increased in the p53 cKO than in the control group for stimuli above 75 μA (Figures 6D–F), suggesting a higher inhibitory status in basal state of p53-cKO.

## 4 Discussion

During neurulation, p53 plays an important role in regulating the proliferation of neuroepithelial cells thus preventing neural tube closure defects. Its loss causes exencephaly in around 37% of p53−/− females, a consequence of overproduction of neural tissue and failure in the neural tube closure (Armstrong et al., 1995; Sah et al., 1995). At the onset of cortical neurogenesis (E11.5), p53 is expressed in proliferative cells (Forsberg et al., 2013), and restrains differentiation of cortical neurons (Forsberg et al., 2013; Liu et al., 2013). However, the role of p53 in programmed cell death is controversial (Miller et al., 2000). To study the role of p53 in cortical and hippocampal formation and function after neural tube closure, we designed a mouse model in which p53 is deleted from glutamatergic progenitors and their derivatives in the pallium as early as E9.5. We did not find any alteration in the cortical thickness or the number of upper or lower cortical neurons, suggesting that either p53 has no effect on neurogenesis and programmed cell death in the pallium or there is a compensatory mechanism by other members of family such as p63 and p73 (Jacobs et al., 2006). DNA damage during brain development causes p53-dependent apoptosis (Xiong et al., 2020). DNA damage could be triggered by targeted mutations in genes encoding for proteins involved in DNA repair (e.g., LIG4, XRCC4, NBN1), microtubule cytoskeleton (e. g. NDE1, CEP63, TUBB5), cell division (e.g., KIF20B, CITK) or RNA metabolism (e.g., EIF4A3, RBM8A, MAGOH) (Frappart et al., 2005; Houlihan and Feng, 2014; Marjanovic et al., 2015; Breuss et al., 2016; Mao et al., 2016; Bianchi et al., 2017; Little and Dwyer, 2019). Mutations in these genes cause apoptosis in the developing cerebral leading to primary microcephaly, and these phenotypes could largely be rescued by p53 deletion. In the adult brain, p53 negatively regulates the proliferation and renewal of neural stem cells (NSCs) of the SVZ (Medrano and Scrable, 2005). Studies using the p53-null mice have shown that the lack of p53 increases the number of progenitors in the SVZ *in vivo* (Gil-Perotin et al., 2006; Meletis et al., 2006) producing more neuroblasts that incorporate in the OB (Gil-Perotin et al., 2011). An increase in olfactory interneurons in the OB in the *p53^−/−^* mice resulted in changes in olfactory behavior but normal memory (Gil-Perotin et al., 2011). Interestingly, 50% reduction of p53 in glutamatergic neurons in conditional *Emx1^Cre^; p53^F/+^* mice impairs social interaction in the three-chamber test, an olfaction-dependent behavior, even though the authors did not discuss whether changes in social behavior are associated with alteration in novel odor exploration (Lee et al., 2023). On the other hand, conditional knock-down mice (*Emx1^Cre^; p53^F/+^*) present impaired hippocampus-dependent learning and memory, a phenotype ascribed to the partial deletion of p53 in pyramidal neurons of the hippocampus (Lee et al., 2023). This is however unlikely since the p53 protein level in the adult brain is maintained very low in neurons by ubiquitination and protein degradation (Jones et al., 1995; Iwakuma and Lozano, 2003). In the cortex and hippocampus, p53 is virtually absent in unstressed control mice, and its expression raises only in response to neuronal injury (Hughes et al., 1996; LaFerla et al., 1996; Sakhi et al., 1996; Morrison and Kinoshita, 2000). To the best of our knowledge, the role of p53 in adult hippocampal neurogenesis has not been addressed. We found that a lack of p53 increases the number of Sox2 progenitors in the dentate subgranular zone and the number of dentate gyrus granule cells. These results are in line with the known role of p53 regulating proliferation in the SVZ, the other neurogenic niche in the adult brain and already discussed previously. Increased adult neurogenesis in the hippocampus has been associated with epileptogenesis (Danzer, 2018). Decreasing granule cell neurogenesis by pharmacological agents or genetic ablation of newborn cells reduces seizure frequency in epileptic mouse models (Jung et al., 2004, 2006; Cho et al., 2015; Hosford et al., 2017). We also found more glutamatergic synapses in the stratum lucidum of the hippocampal CA3, the region where granule cells connect with CA3 pyramidal neurons. The rise in glutamatergic synapses in p53-cKO mice does not trigger overexcitability in basal conditions in the CA3 region, most probably due to a compensatory mechanism by the increase of inhibitory synapses. When GABAA receptors are blocked by picrotoxin, the mutant hippocampus presents higher postsynaptic responses, confirming the augmentation of the inhibitory status in basal condition. Granule cells in the dentate gyrus send their axons to the CA3 region of the hippocampus where they make powerful excitatory “detonator” synapses on CA3 pyramidal neurons (Henze et al., 2002). An increase in granule cell activation is associated with epilepsy development (Dengler et al., 2017). We also found that granule cells are spread in the superior granule cell layer in p53-cKO mice, suggesting a migration defect in a non-cell-autonomous manner. One of the factors implicated in the migration of granule cells in the hippocampus is the extracellular protein reelin released by Cajal–Retzius cells (Wang et al., 2018). These cells in the hippocampus persist in the postnatal brain and they are localized in the outer molecular layer of the dentate gyrus (Anstotz et al., 2016, 2018). Interestingly, the epileptogenic hippocampus was associated with a decrease in reelin signaling in humans and mice (Haas et al., 2002; Gong et al., 2007) suggesting that abnormal spreading granule cells in the p53-cKO mice could be a consequence of reelin downregulation due to seizure episodes.

Overall, this study shed light on the functional consequences of p53 deletion in cortical and hippocampal development and function. p53 is dispensable for programmed cell death in the developing cortex but essential to eliminating dysfunctional cells or cells with high oxidative stress, DNA damage, or aneuploidy. Indeed, karyotype aberrations are associated with cancer development (Davoli et al., 2013) and the inactivation of p53 is linked with the development of several cancers. Interestingly, p53 is commonly mutated in glioblastoma (GBM) (Li et al., 2023), the most severe primary malignant tumor of the adult brain. NSCs of the SVZ are believed to be one of the cells of origin for GBM in humans (Beiriger et al., 2022). Given that p53 has a role in NSCs proliferation and self-renewal in the SVZ, its implication in glioblastoma genesis deserves further investigations.

## Data availability statement

The original contributions presented in the study are included in the article/Supplementary material, further inquiries can be directed to the corresponding authors.

## Ethics statement

The animal study was approved by the animal ethics committee of the Université Catholique de Louvain under agreement 2019/UCL/MD/006. The study was conducted in accordance with the local legislation and institutional requirements.

## Author contributions

NR-R: Conceptualization, Formal analysis, Investigation, Methodology, Supervision, Validation, Writing – original draft, Writing – review & editing, Data curation, Funding acquisition. GC: Data curation, Formal analysis, Investigation, Methodology, Validation, Writing – review & editing. XY: Formal Analysis, Investigation, Methodology, Writing – review & editing. ID: Data curation, Formal analysis, Investigation, Validation, Writing – review & editing. PG: Conceptualization, Formal analysis, Writing – review & editing. FT: Conceptualization, Formal analysis, Funding acquisition, Resources, Supervision, Writing – original draft, Writing – review & editing.

## References

[ref1] Anderson W. W. Collingridge G. L. (2007). Capabilities of the WinLTP data acquisition program extending beyond basic LTP experimental functions. J. Neurosci. Methods 162, 346–356. doi: 10.1016/j.jneumeth.2006.12.018, PMID: 17306885

[ref2] Anstotz M. Huang H. Marchionni I. Haumann I. Maccaferri G. Lubke J. H. (2016). Developmental profile, morphology, and synaptic connectivity of Cajal-Retzius cells in the postnatal mouse Hippocampus. Cereb. Cortex 26, 855–872. doi: 10.1093/cercor/bhv271, PMID: 26582498 PMC4712808

[ref3] Anstotz M. Quattrocolo G. Maccaferri G. (2018). Cajal-Retzius cells and GABAergic interneurons of the developing hippocampus: close electrophysiological encounters of the third kind. Brain Res. 1697, 124–133. doi: 10.1016/j.brainres.2018.07.028, PMID: 30071194 PMC6093789

[ref4] Armesilla-Diaz A. Bragado P. Del Valle I. Cuevas E. Lazaro I. Martin C.. (2009). p53 regulates the self-renewal and differentiation of neural precursors. Neuroscience 158, 1378–1389. doi: 10.1016/j.neuroscience.2008.10.052, PMID: 19038313

[ref5] Armstrong J. F. Kaufman M. H. Harrison D. J. Clarke A. R. (1995). High-frequency developmental abnormalities in p53-deficient mice. Curr. Biol. 5, 931–936. doi: 10.1016/s0960-9822(95)00183-7, PMID: 7583151

[ref6] Beiriger J. Habib A. Jovanovich N. Kodavali C. V. Edwards L. Amankulor N.. (2022). The subventricular zone in glioblastoma: genesis, maintenance, and modeling. Front. Oncol. 12:790976. doi: 10.3389/fonc.2022.790976, PMID: 35359410 PMC8960165

[ref7] Bianchi F. T. Tocco C. Pallavicini G. Liu Y. Verni F. Merigliano C.. (2017). Citron kinase deficiency leads to chromosomal instability and TP53-sensitive microcephaly. Cell Rep. 18, 1674–1686. doi: 10.1016/j.celrep.2017.01.054, PMID: 28199840 PMC5318669

[ref8] Breuss M. Fritz T. Gstrein T. Chan K. Ushakova L. Yu N.. (2016). Mutations in the murine homologue of TUBB5 cause microcephaly by perturbing cell cycle progression and inducing p53-associated apoptosis. Development 143, 1126–1133. doi: 10.1242/dev.131516, PMID: 26903504

[ref9] Chipuk J. E. Kuwana T. Bouchier-Hayes L. Droin N. M. Newmeyer D. D. Schuler M.. (2004). Direct activation of Bax by p53 mediates mitochondrial membrane permeabilization and apoptosis. Science 303, 1010–1014. doi: 10.1126/science.1092734, PMID: 14963330

[ref10] Cho K. O. Lybrand Z. R. Ito N. Brulet R. Tafacory F. Zhang L.. (2015). Aberrant hippocampal neurogenesis contributes to epilepsy and associated cognitive decline. Nat. Commun. 6:6606. doi: 10.1038/ncomms7606, PMID: 25808087 PMC4375780

[ref11] Danzer S. C. (2018). Contributions of adult-generated granule cells to hippocampal pathology in temporal lobe epilepsy: a neuronal bestiary. Brain Plast 3, 169–181. doi: 10.3233/BPL-170056 30151341 PMC6091048

[ref12] Davoli T. Xu A. W. Mengwasser K. E. Sack L. M. Yoon J. C. Park P. J.. (2013). Cumulative haploinsufficiency and triplosensitivity drive aneuploidy patterns and shape the cancer genome. Cell 155, 948–962. doi: 10.1016/j.cell.2013.10.011, PMID: 24183448 PMC3891052

[ref13] Dengler C. G. Yue C. Takano H. Coulter D. A. (2017). Massively augmented hippocampal dentate granule cell activation accompanies epilepsy development. Sci. Rep. 7:42090. doi: 10.1038/srep42090, PMID: 28218241 PMC5316990

[ref14] Di Giovanni S. Knights C. D. Rao M. Yakovlev A. Beers J. Catania J.. (2006). The tumor suppressor protein p53 is required for neurite outgrowth and axon regeneration. EMBO J. 25, 4084–4096. doi: 10.1038/sj.emboj.7601292, PMID: 16946709 PMC1560361

[ref15] Forsberg K. Wuttke A. Quadrato G. Chumakov P. M. Wizenmann A. Di Giovanni S. (2013). The tumor suppressor p53 fine-tunes reactive oxygen species levels and neurogenesis via PI3 kinase signaling. J. Neurosci. 33, 14318–14330. doi: 10.1523/JNEUROSCI.1056-13.2013, PMID: 24005285 PMC6618388

[ref16] Frappart P. O. Tong W. M. Demuth I. Radovanovic I. Herceg Z. Aguzzi A.. (2005). An essential function for NBS1 in the prevention of ataxia and cerebellar defects. Nat. Med. 11, 538–544. doi: 10.1038/nm1228 15821748

[ref17] Gil-Perotin S. Haines J. D. Kaur J. Marin-Husstege M. Spinetta M. J. Kim K. H.. (2011). Roles of p53 and p27(Kip1) in the regulation of neurogenesis in the murine adult subventricular zone. Eur. J. Neurosci. 34, 1040–1052. doi: 10.1111/j.1460-9568.2011.07836.x, PMID: 21899604 PMC3214596

[ref18] Gil-Perotin S. Marin-Husstege M. Li J. Soriano-Navarro M. Zindy F. Roussel M. F.. (2006). Loss of p53 induces changes in the behavior of subventricular zone cells: implication for the genesis of glial tumors. J. Neurosci. 26, 1107–1116. doi: 10.1523/JNEUROSCI.3970-05.2006, PMID: 16436596 PMC6674560

[ref19] Gong C. Wang T. W. Huang H. S. Parent J. M. (2007). Reelin regulates neuronal progenitor migration in intact and epileptic hippocampus. J. Neurosci. 27, 1803–1811. doi: 10.1523/JNEUROSCI.3111-06.2007, PMID: 17314278 PMC6673551

[ref20] Gorski J. A. Talley T. Qiu M. Puelles L. Rubenstein J. L. Jones K. R. (2002). Cortical excitatory neurons and glia, but not GABAergic neurons, are produced in the Emx1-expressing lineage. J. Neurosci. 22, 6309–6314. doi: 10.1523/JNEUROSCI.22-15-06309.2002, PMID: 12151506 PMC6758181

[ref21] Haas C. A. Dudeck O. Kirsch M. Huszka C. Kann G. Pollak S.. (2002). Role for reelin in the development of granule cell dispersion in temporal lobe epilepsy. J. Neurosci. 22, 5797–5802. doi: 10.1523/JNEUROSCI.22-14-05797.2002, PMID: 12122039 PMC6757930

[ref22] Henze D. A. Wittner L. Buzsaki G. (2002). Single granule cells reliably discharge targets in the hippocampal CA3 network in vivo. Nat. Neurosci. 5, 790–795. doi: 10.1038/nn887, PMID: 12118256

[ref23] Hosford B. E. Rowley S. Liska J. P. Danzer S. C. (2017). Ablation of peri-insult generated granule cells after epilepsy onset halts disease progression. Sci. Rep. 7:18015. doi: 10.1038/s41598-017-18237-6, PMID: 29269775 PMC5740143

[ref24] Houlihan S. L. Feng Y. (2014). The scaffold protein Nde1 safeguards the brain genome during S phase of early neural progenitor differentiation. eLife 3:e03297. doi: 10.7554/eLife.03297, PMID: 25245017 PMC4170211

[ref25] Hughes P. E. Alexi T. Yoshida T. Schreiber S. S. Knusel B. (1996). Excitotoxic lesion of rat brain with quinolinic acid induces expression of p53 messenger RNA and protein and p53-inducible genes Bax and Gadd-45 in brain areas showing DNA fragmentation. Neuroscience 74, 1143–1160. doi: 10.1016/0306-4522(96)00174-1, PMID: 8895882

[ref26] Iwakuma T. Lozano G. (2003). MDM2, an introduction. Mol. Cancer Res. 1, 993–1000., PMID: 14707282

[ref27] Jacobs W. B. Kaplan D. R. Miller F. D. (2006). The p53 family in nervous system development and disease. J. Neurochem. 97, 1571–1584. doi: 10.1111/j.1471-4159.2006.03980.x 16805769

[ref28] Jones S. N. Roe A. E. Donehower L. A. Bradley A. (1995). Rescue of embryonic lethality in Mdm2-deficient mice by absence of p53. Nature 378, 206–208. doi: 10.1038/378206a0 7477327

[ref29] Jung K. H. Chu K. Kim M. Jeong S. W. Song Y. M. Lee S. T.. (2004). Continuous cytosine-b-D-arabinofuranoside infusion reduces ectopic granule cells in adult rat hippocampus with attenuation of spontaneous recurrent seizures following pilocarpine-induced status epilepticus. Eur. J. Neurosci. 19, 3219–3226. doi: 10.1111/j.0953-816X.2004.03412.x, PMID: 15217378

[ref30] Jung K. H. Chu K. Lee S. T. Kim J. Sinn D. I. Kim J. M.. (2006). Cyclooxygenase-2 inhibitor, celecoxib, inhibits the altered hippocampal neurogenesis with attenuation of spontaneous recurrent seizures following pilocarpine-induced status epilepticus. Neurobiol. Dis. 23, 237–246. doi: 10.1016/j.nbd.2006.02.016, PMID: 16806953

[ref31] LaFerla F. M. Hall C. K. Ngo L. Jay G. (1996). Extracellular deposition of beta-amyloid upon p53-dependent neuronal cell death in transgenic mice. J. Clin. Invest. 98, 1626–1632. doi: 10.1172/JCI118957, PMID: 8833912 PMC507596

[ref32] Lee K. Y. Wang H. Yook Y. Rhodes J. S. Christian-Hinman C. A. Tsai N. P. (2023). Tumor suppressor p53 modulates activity-dependent synapse strengthening, autism-like behavior and hippocampus-dependent learning. Mol. Psychiatry 28, 3782–3794. doi: 10.1038/s41380-023-02268-9, PMID: 37759036 PMC11392564

[ref33] Lepannetier S. Gualdani R. Tempesta S. Schakman O. Seghers F. Kreis A.. (2018). Activation of TRPC1 channel by metabotropic glutamate receptor mGluR5 modulates synaptic plasticity and spatial working memory. Front. Cell. Neurosci. 12:318. doi: 10.3389/fncel.2018.00318, PMID: 30271326 PMC6149316

[ref34] Leu J. I. Dumont P. Hafey M. Murphy M. E. George D. L. (2004). Mitochondrial p53 activates Bak and causes disruption of a Bak-Mcl1 complex. Nat. Cell Biol. 6, 443–450. doi: 10.1038/ncb1123, PMID: 15077116

[ref35] Li H. Zhang Z. Li H. Pan X. Wang Y. (2023). New insights into the roles of p53 in central nervous system diseases. Int. J. Neuropsychopharmacol. 26, 465–473. doi: 10.1093/ijnp/pyad030, PMID: 37338366 PMC10388388

[ref36] Lindsten T. Ross A. J. King A. Zong W. X. Rathmell J. C. Shiels H. A.. (2000). The combined functions of proapoptotic Bcl-2 family members bak and bax are essential for normal development of multiple tissues. Mol. Cell 6, 1389–1399. doi: 10.1016/s1097-2765(00)00136-2, PMID: 11163212 PMC3057227

[ref37] Little J. N. Dwyer N. D. (2019). p53 deletion rescues lethal microcephaly in a mouse model with neural stem cell abscission defects. Hum. Mol. Genet. 28, 434–447. doi: 10.1093/hmg/ddy350, PMID: 30304535 PMC6337704

[ref38] Liu H. Jia D. Li A. Chau J. He D. Ruan X.. (2013). p53 regulates neural stem cell proliferation and differentiation via BMP-Smad1 signaling and Id1. Stem Cells Dev. 22, 913–927. doi: 10.1089/scd.2012.0370, PMID: 23199293 PMC3585476

[ref39] Mao H. McMahon J. J. Tsai Y. H. Wang Z. Silver D. L. (2016). Haploinsufficiency for Core exon junction complex components disrupts embryonic neurogenesis and causes p53-mediated microcephaly. PLoS Genet. 12:e1006282. doi: 10.1371/journal.pgen.1006282, PMID: 27618312 PMC5019403

[ref40] Marino S. Vooijs M. van Der Gulden H. Jonkers J. Berns A. (2000). Induction of medulloblastomas in p53-null mutant mice by somatic inactivation of Rb in the external granular layer cells of the cerebellum. Genes Dev. 14, 994–1004. doi: 10.1101/gad.14.8.994, PMID: 10783170 PMC316543

[ref41] Marjanovic M. Sanchez-Huertas C. Terre B. Gomez R. Scheel J. F. Pacheco S.. (2015). CEP63 deficiency promotes p53-dependent microcephaly and reveals a role for the centrosome in meiotic recombination. Nat. Commun. 6:7676. doi: 10.1038/ncomms8676, PMID: 26158450 PMC4499871

[ref42] Medrano S. Scrable H. (2005). Maintaining appearances--the role of p53 in adult neurogenesis. Biochem. Biophys. Res. Commun. 331, 828–833. doi: 10.1016/j.bbrc.2005.03.194 15865938

[ref43] Meletis K. Wirta V. Hede S. M. Nister M. Lundeberg J. Frisen J. (2006). p53 suppresses the self-renewal of adult neural stem cells. Development 133, 363–369. doi: 10.1242/dev.02208, PMID: 16368933

[ref44] Miller F. D. Pozniak C. D. Walsh G. S. (2000). Neuronal life and death: an essential role for the p53 family. Cell Death Differ. 7, 880–888. doi: 10.1038/sj.cdd.4400736, PMID: 11279533

[ref45] Morrison R. S. Kinoshita Y. (2000). The role of p53 in neuronal cell death. Cell Death Differ. 7, 868–879. doi: 10.1038/sj.cdd.4400741 11279532

[ref46] Ruiz-Reig N. Andres B. Huilgol D. Grove E. A. Tissir F. Tole S.. (2017). Lateral thalamic Eminence: a novel origin for mGluR1/lot cells. Cereb. Cortex 27, bhw126–bhw2856. doi: 10.1093/cercor/bhw126, PMID: 27178193 PMC6248457

[ref47] Ruiz-Reig N. Andres B. Lamonerie T. Theil T. Fairen A. Studer M. (2018). The caudo-ventral pallium is a novel pallial domain expressing Gdf10 and generating Ebf3-positive neurons of the medial amygdala. Brain Struct. Funct. 223, 3279–3295. doi: 10.1007/s00429-018-1687-0, PMID: 29869132

[ref48] Ruiz-Reig N. Chehade G. Hakanen J. Aittaleb M. Wierda K. De Wit J.. (2022). KIF2A deficiency causes early-onset neurodegeneration. Proc. Natl. Acad. Sci. USA 119:e2209714119. doi: 10.1073/pnas.2209714119, PMID: 36343267 PMC9674219

[ref49] Ruiz-Reig N. Hakanen J. Tissir F. (2024). Connecting neurodevelopment to neurodegeneration: a spotlight on the role of kinesin superfamily protein 2A (KIF2A). Neural Regen. Res. 19, 375–379. doi: 10.4103/1673-5374.375298, PMID: 37488893 PMC10503618

[ref50] Sah V. P. Attardi L. D. Mulligan G. J. Williams B. O. Bronson R. T. Jacks T. (1995). A subset of p53-deficient embryos exhibit exencephaly. Nat. Genet. 10, 175–180. doi: 10.1038/ng0695-175, PMID: 7663512

[ref51] Sakhi S. Sun N. Wing L. L. Mehta P. Schreiber S. S. (1996). Nuclear accumulation of p53 protein following kainic acid-induced seizures. Neuroreport 7, 493–496. doi: 10.1097/00001756-199601310-00028, PMID: 8730813

[ref52] Schmid P. Lorenz A. Hameister H. Montenarh M. (1991). Expression of p53 during mouse embryogenesis. Development 113, 857–865. doi: 10.1242/dev.113.3.857 1821855

[ref53] Shimada T. Yamagata K. (2018). Pentylenetetrazole-induced kindling mouse model. J. Vis. Exp. 136, 1–10. doi: 10.3791/56573, PMID: 29985308 PMC6101698

[ref54] Sun C. N. Chuang H. C. Wang J. Y. Chen S. Y. Cheng Y. Y. Lee C. F.. (2010). The A2A adenosine receptor rescues neuritogenesis impaired by p53 blockage via KIF2A, a kinesin family member. Dev. Neurobiol. 70, 604–621. doi: 10.1002/dneu.20802, PMID: 20506231

[ref55] Tedeschi A. Nguyen T. Puttagunta R. Gaub P. Di Giovanni S. (2009). A p53-CBP/p300 transcription module is required for GAP-43 expression, axon outgrowth, and regeneration. Cell Death Differ. 16, 543–554. doi: 10.1038/cdd.2008.175, PMID: 19057620

[ref56] van den Berghe V. Stappers E. Vandesande B. Dimidschstein J. Kroes R. Francis A.. (2013). Directed migration of cortical interneurons depends on the cell-autonomous action of Sip1. Neuron 77, 70–82. doi: 10.1016/j.neuron.2012.11.009, PMID: 23312517

[ref57] van Lookeren Campagne M. Gill R. (1998). Tumor-suppressor p53 is expressed in proliferating and newly formed neurons of the embryonic and postnatal rat brain: comparison with expression of the cell cycle regulators p21Waf1/Cip1, p27Kip1, p57Kip2, p16Ink4a, cyclin G1, and the proto-oncogene Bax. J. Comp. Neurol. 397, 181–198. doi: 10.1002/(sici)1096-9861(19980727)397:2<181::aid-cne3>3.0.co;2-x, PMID: 9658283

[ref58] Wang S. Brunne B. Zhao S. Chai X. Li J. Lau J.. (2018). Trajectory analysis unveils Reelin's role in the directed migration of granule cells in the dentate gyrus. J. Neurosci. 38, 137–148. doi: 10.1523/JNEUROSCI.0988-17.2017, PMID: 29138282 PMC6705807

[ref59] Wei M. C. Zong W. X. Cheng E. H. Lindsten T. Panoutsakopoulou V. Ross A. J.. (2001). Proapoptotic BAX and BAK: a requisite gateway to mitochondrial dysfunction and death. Science 292, 727–730. doi: 10.1126/science.1059108, PMID: 11326099 PMC3049805

[ref60] Wong F. K. Marin O. (2019). Developmental cell death in the cerebral cortex. Annu. Rev. Cell Dev. Biol. 35, 523–542. doi: 10.1146/annurev-cellbio-100818-125204 31283379

[ref61] Xiong Y. Zhang Y. Xiong S. Williams-Villalobo A. E. (2020). A glance of p53 functions in brain development, neural stem cells, and brain Cancer. Biology 9, 1–13. doi: 10.3390/biology9090285, PMID: 32932978 PMC7564678

[ref62] Yamaguchi Y. Miura M. (2015). Programmed cell death in neurodevelopment. Dev. Cell 32, 478–490. doi: 10.1016/j.devcel.2015.01.019 25710534

[ref63] Yerna X. Schakman O. Ratbi I. Kreis A. Lepannetier S. de Clippele M.. (2020). Role of the TRPC1 channel in hippocampal long-term depression and in spatial memory extinction. Int. J. Mol. Sci. 21, 1–13. doi: 10.3390/ijms21051712, PMID: 32138218 PMC7084652

