# Supplementary material for: Aberrant generation of dentate gyrus granule cells is associated with epileptic susceptibility in p53 conditional knockout mice
Source: Front Neurosci. 2024 Aug 14;18:1418973. doi: 10.3389/fnins.2024.1418973 (PMC11349535; doi:10.3389/fnins.2024.1418973)
Supplement: Supplementary file 1 [file Data_Sheet_1.PDF]

Supplementary Material

1 Supplementary Data

Movie S1. Spontaneous seizures in p53-cKO mice. Video showing spontaneous seizures in two out of three p53-cKO mice at 2 months of age. Mice were housed in standard conditions at the Université catholique de Louvain.

Movie S2. PTZ-induced seizure in p53-cKO mice. Monitoring of control and p53-cKO mice during the PTZ-post injection period. The p53 cKO mouse (right) exhibits a tonic-clonic seizure (score 3: continuous whole-body myoclonus, myoclonic jerks, tail held up stiffly), while the control littermate (left) is unaffected (score 0).
